# Medical Association of Central New York

**Published:** 1906-06

**Authors:** C. A. Greenleaf

**Affiliations:** Secretary, of Rochester; Buffalo


### SOCIETY PROCEEDINGS.

### Medical Association of Central New York.

(*Continued from page 600, May.*)

Reported by Dr. C. A. GREENLEAF, Secretary, of Rochester.

(Edited by William C. Krauss, M. D., Buffalo.)

### AFTERNOON SESSION.

Afternoon session called to order at three o'clock.

The president: Nominations for officers for the ensuing year should come up at this time.

Wm. C. Krauss, Buffalo, moved that the Association proceed with the election of officers for the ensuing year.

Seconded and carried.

H. L. Elsner, Syracuse: Mr. President, Gentlemen of the Association: Our present Vice-president we all know is one of the most enthusiastic, earnest and willing workers in the Association, and without taking your time I would suggest that the nomination of Dr. David M. Totman of Syracuse for President for the ensuing year be made unanimous.

A. A. Young, Newark, seconded the nomination.

The nomination was voted upon and carried unanimously. The President declared Dr. Totman duly elected President for the ensuing year.

The president: Nominations are in order for the office of first vice-president.

N. Jacobson, Syracuse: The custom has been to select a vice-president from the place at which the second meeting would be held; consequently the selection of the vice-president should be accorded to the city of Rochester. For that reason it gives me pleasure to put in nomination the name of Dr. William B. Jones of Rochester.

Nomination seconded.

The nomination was voted upon and carried unanimously. The president declared Dr. Jones duly elected first vice-president for the ensuing year.

The president: There is also to be elected the second vice-president.

Wm. C. Krauss, Buffalo: I nominate for the office of second vice-president a physician who has been active in practice in Western New York for many years, and who is with us today with a goodly gathering from Genesee county, Dr. L. L. Tozier.

Nomination seconded.

The nomination was voted upon and carried unanimously. The president declared Dr. Tozier duly elected second vice-president for the ensuing year.

W. C. Krauss, Buffalo: For the Committee of Arrangements I wish to announce that tonight at seven o'clock we expect you all to be present at the Buffalo Club. Dinner will follow at seven-thirty, but it is desirable to have you there promptly at seven, because some are obliged to leave on the nine o'clock train. The Buffalo Club is at 388 Delaware Avenue, nearly opposite Tupper street, and the Committee will be pleased to see every body present.

The first paper of the afternoon is "Preliminary Report on the Therapeutic Value of Antithyroidine in the Treatment of Exophthalmic Goiter and Kindred Affections," by Dr. Henry L. Elsner and Joseph R. Wiseman of Syracuse.

Delancey Rochester, Buffalo: This appeals to me very much, because I have had a slight experience with a similar preparation, a preparation made from the blood of thyroid of sheep by Parke, Davis & Company, which they call Thyroidaxine. I have had three cases under observation for about a year, and two of them have disappeared from view in the last two months, apparently cured as far as any subjective symptoms are concerned. In these cases the thyroid, still remains enlarged, as in Dr. Elsner's. One case was referred to me last winter of a very extreme degree of Grave's disease, the patient was suffering very much. She improved very materially, and then by mistake of one of the druggists he gave her thyroid extract instead of the capsules and the symptoms were greatly increased. She came back in a terrible state because the druggist gave it to her in the form of tablet instead of capsule. Then she improved, and last May she disappeared from view, but came back to me the first part of this month saying that she had remained improved and very comfortable for a couple of months. About the middle of August she began to have the symptoms again, distressing her very much. She returned and I put her on the drug again the first of October and she was in to see me this morning, feeling a hundred per cent. better, as she expressed it to me, and certainly appeared so. I simply want to state these cases to Dr. Elsner as improved.

W. D. JOHNSON, Batavia: There is one suggestion that occurs to me that might be of practical interest to the surgeon who wanted to remove the thyroid in exophthalmic goiter; that he might prepare his patient for the operation and avoid the acute thyroidism that sometimes occurs from handling the glands, by preparing the patient with Antithyroidine, and thereby prevent the overwhelming of the system by the excess of thyroid secretion that he throws in the system during manipulation. It may be of some interest to surgeons.

DELANCEY ROCHESTER, Buffalo, read a paper entitled "Cholecystitis."

DISCUSSION.

H. L. ELSNER, Syracuse: I do not feel that this paper ought to go without a word of discussion, and I wish to report a case in connection with the cases reported by Dr. Rochester which we recently saw at Oswego with Dr. Eddy in consultation, proving the correctness of Dr. Rochester's teachings. It was the case of a young woman who was in the fourth or fifth week of typhoid fever, and who had a rather interesting early history, showing the tolerance of some people. She was at St. Louis attending the fair. She crossed the country from St. Louis to Boston in an automobile, during which time she was in the early stages of typhoid. She came to Oswego in, I think, the third week of typhoid. Towards the end of the fourth week she developed severe pains in the region of the gall bladder and showed particularly the symptoms of cholecystitis and infected gall bladder and gall-stones.

The history was very vague. The woman did not remember having had attacks of gallstone. We searched in every possible way to get some clue as to possible gallstone colic, until finally her husband volunteered the information that when she was engaged to be married he remembered on night going out for a physician to come in to give her a hypodermic injection for a severe pain which she had in her abdomen, and that was I think about fifteen years before this typhoid and infected gall bladder. We thereupon made the diagnosis of gall stone cholecystitis, typhoid infection, and recommended operation. The case was operated by Dr. Jacobson. I do not remember just how many gall-stones we found, but we found a great number, an infected gall bladder, and fortunately this woman has made a perfect recovery. The bacteriological examination made proved the presence of the typhoid. We are perfectly satisfied that without the operation this woman would have died. We had several cases which ran about parallel with this, which prove the correctness of this teaching, which I think is a lesson which we should take home with us, that an infected gall bladder needs drainage, which needs relief, and there will always be a condition there which continues to be dangerous until it is relieved.

A. L. Benedict, Buffalo: I told Dr. Rochester in jest that I agreed so thoroughly with his interesting paper that I disagreed with him, but only in this way, that I am inclined to be a little credulous of the action of salicylate and hot water and treatment of that sort, and which is supposed to dissolve gall-stones. It certainly does not very often, but it sometimes expedites the passage of the stones, and I am old-fashioned enough to stand here and say that I would a great deal rather be alive with gall-stones in me than dead with them out. I remember a case where four of us made a diagnosis of gall-stone. It was absolutely a typical case if we ever saw one of gall-stone. My advice to the patient was to try medical treatment persistently for a couple of months and then if not relieved to resort to the operaion, which he had already been advised to have. He tried the treatment in a desultory way for a couple of weeks, and finally had the operation, but the gall bladder was found empty. I think if there were gall-stones present he had passed them.

To save the time later in the program I would like to pass around this stool sieve. It is so arranged that a flow of water can very easily be obtained, and by setting this in a sink there is very little soiling of the wash-basin. I would like to pass the stomach tube also. I find it is perfectly easy to get the stomach contents with a good sized tube, which I cannot do with a small one, and this tube will work very nicely in the average esophagus and will save time.

N. Jacobson, Syracuse: This is an important matter, and I think the point Dr. Rochester missed is one that ought to be emphasised. When you have to deal with a septic condition of the gall-bladder I do not know of any way of clearing up that condition except by drainage. Gall-stones may lie in the bladder a lifetime and cause no trouble, but when you are dealing with cholecystitis then I feel we must drain. I disagree with the writer of the paper that it is not a good idea to operate until your septic condition is controlled. We often get associated with gall-stones a mass of bile that is passed, it looks like a lot of gall-stones and stuff that passes, and the size of your gall bladder may be reduced, the contents of the bladder, the septic contents may pass off, because this has been plugging up the gall outlets rather than the stones. In one particular case I have in mind large masses of thickened bile were passed. We meet cholecystitis where you don't get any septic condition. I have had a number of cases where I expected to find considerable septic material and I did not find anything but a lot of gall-stones or thick bile that filled up the gall bladder and plugged up the cystic duct. In all of my cases of cholecystitis—and there are quite a number now—I have not seen one die when he was operated on. The only fatalities I have had are those cases that come to us so late. In those cases I have seen two deaths. In the others I have seen none. This condition is a thing not to be overlooked and it is a thing not to be neglected.

John O. Roe, Rochester, read a paper entitled "Vibratory and Vapor Massage in the Treatment of Diseases of the Ear and Upper Air Passages."

Nathan Jacobson, Syracuse, read a paper entitled "Surgery of the Prostate."

M. B. Tinker, Clifton Springs: I would like to say in connection with Dr. Jacobson's paper, that in certain very bad cases, in certain cases where you have to deal with an old man greatly reduced, where the function of the kidney is doubtful, Dr. Young has been accustomed to use supra-pubic prostatectomy, to drain the bladder a short time until he can determine whether the patient will stand the greater operation. I would also like to say that I have on three occasions done a perineal prostatectomy successfully under local anesthesia, and in at least two of the cases feel that with the lessened shock of local anesthesia one is able to save lives of patients who otherwise would have died under general anesthesia. In regard to local anesthesia, I would say that those of you who remember the anatomy of the perineum recall that the nerve supply comes from two nerve trunks that wind around the base of the tuberosity of the ischium. I was able to study the location of the nerves in all the dissections during one term, and I found that you can usually reach the nerve with a long needle and inject directly into it. In two of the cases I succeeded in reaching the nerve and getting good anesthesia, and in one I failed. Although I used infiltration in connection with it there was a considerable degree of pain. I believe in these cases if you have become accustomed to the use of local anesthesia it is possible to do supra-pubic prostatectomy very satisfactorily undr local anesthesia, and to save the lives of men who would not stand the general anesthesia, although I would say that I have seen in my own practice, and in that of others, men of eighty years take the general anesthesia for prostatectomy without bad results, and very many of these hopeless cases will recover if given the benefit of this operation and drainage of the bladder.

DR. LOEB: This seems to be an important question, and it is one which is brought before us prominently today. It is an operation which brings relief which the general practitioner cannot give. We all have old men with prostatitis, we all know that sooner or later he gets an infection of the bladder and it extends to the kidney and it uses him up. If there is any relief from early operation it cannot be too forcibly carried to the mind of the general practitioner that he should have this condition under constant observation and turned over to the hands of the surgeon while the case is yet susceptible of treatment.

The president: Before we pass to the next paper, it has been brought to my attention that we have met the loss in our Association of one of the founders of the Association, the late Dr. Didama of Syracuse, and it seems to me it would be very proper for us to take some action in relation to his demise.

E. B. ANGELL, Rochester: I move that a committee be appointed by the Chair to draw up a set of resolutions on the death of Dr. Didama and that it be reported to the Secretary and spread upon the minutes, unless they can report in time to pass upon it this afternoon.

Motion seconded and carried.

The President appointed Dr. Elsner of Syracuse, Dr. Angell of Rochester, and Dr. Tozier of Batavia.

E. S. VANDUYN, of Syracuse, read a paper entitled "The Question of Local and General Anesthesia."

EDWARD FLAHERTY, of Syracuse, read a paper entitled "Report of Case: Operation for Strangulated Femoral Hernia in a Woman 95 Years Old, under Local Anesthesia."

M. D. MANN, Buffalo: I have done a number of small operations with local anesthesia. On Friday I was in Chicago and in a clinic with Dr. Webster I saw an exceedingly interesting case. A woman was suffering from a large hernia and her kidneys were seriously diseased. The doctor was afraid to give her a general anesthetic. He injected all the tissues around the place, took out the tubes and ovaries, loosened up the adhesions, cauterised the wound, and sewed the uterus over. The whole operation was very long, an hour and a quarter or a half, I don't remember exactly, and without any anesthesia except once or twice he used chloroform, just a little to quiet her nervousness, due to the length of the operation. I saw the patient the next day and asked her whether it hurt her very much, and she said no, there was scarcely any pain at all. It was a long operation and she got rather tired and nervous, but there was no severe pain, and this is rather remarkable. It was very instructive to me, and I certainly think if occasion requires I should have little or no hesitation in using cocaine for local anesthesia.

(*Concluded next month.*)

